# Genome Guided Personalized Drug Therapy in Attention Deficit Hyperactivity Disorder

**DOI:** 10.3389/fpsyt.2022.925442

**Published:** 2022-06-27

**Authors:** Jan Haavik

**Affiliations:** ^1^Department of Biomedicine, University of Bergen, Bergen, Norway; ^2^Bergen Center of Brain Plasticity, Division of Psychiatry, Haukeland University Hospital, Bergen, Norway

**Keywords:** ADHD, genetics, exome sequencing, tyrosinemia, pharmacology, neurometabolic diseases, precision medicine, phenylketonuria (PKU)

## Abstract

ADHD is a common behavioral syndrome with a heritability of 70–80%. Genome wide sequencing and association studies indicate that ADHD risk variants are distributed across a wide range of allele frequencies and relative risks. Several common single nucleotide variants (SNPs) have been identified that increase the risk of ADHD with a few percent. Many of the reported risk genes and copy number variants are shared with other neuropsychiatric disorders. Moreover, ADHD often coexists with common or rare somatic diseases, including rare Mendelian neurometabolic diseases that can affect normal brain development and function. Some genetic/metabolic syndromes masquerading as common ADHD may lead to irreversible brain damage if not properly identified and treated during early childhood. As ADHD is such a heterogeneous condition in terms of severity, clinical features and most probably also underlying biology, it is crucial to offer individualized treatments. Recent progress in ADHD genetics is reviewed, prospects of using this information for targeted pharmacotherapy are discussed and critical knowledge gaps are identified. It is suggested that genome guided therapies could be introduced gradually, starting with rare ADHD syndromes with highly penetrant risk genes. Routine diagnostic application of whole exome or whole genome sequencing combined with metabolomic screening, and brain imaging may be needed in cases with suspected neurometabolic disorders. Identification and treatment of ADHD patients with defined neurometabolic aberrations could be a first step toward genome guided personalized treatment of ADHD. Possibly, screening for relevant biomarkers may gradually be implemented to guide treatment choices in larger patient groups.

## Introduction

Attention-deficit/hyperactivity disorder (ADHD) is defined a childhood onset disorder with behavioral symptoms and problems that interfere with normal development and functions (1). It is considered a complex clinical syndrome with multiple risk factors and etiologies that are broadly classified as environmental or genetic in origin. Patient should be diagnosed with ADHD only if the symptoms are not better explained by another disorder (2). However, as the exact etiology of ADHD symptoms in most cases is unknown, it is recommended to diagnose ADHD also in the presence of other psychiatric or somatic symptoms or conditions. This practice has resulted in high reported rates of comorbidity in adult and child ADHD clinical samples and research settings.

ADHD genetics has been intensively investigated and subject to multiple reviews (1, 3, 4). Early family, twin, and adoption studies clearly demonstrated that ADHD runs in families. Based on many studies comparing ADHD symptoms in monozygotic and dizygotic twins, a heritability around 70–80% (mean 74%) has been calculated (3). This implies that environmental factors also are important. Several such environmental risk factors, including birth complications, exposure to environmental toxins, or dietary factors have been suggested, but their relative importance and causal roles are still being debated (4). Environmental exposures are probably interacting with genetic factors and may be important during critical developmental stages either before or after birth (5). Conventional molecular genetic technologies are currently being supplemented by epigenetic studies and other biomarker analyses. Such studies are also used to explore genetic interactions with environmental exposures (G × E interactions), treatment response, and can also reveal parent of origin effects. Parent of origin effects are obvious in several rare genetic syndromes but are probably also important in complex conditions. G × E interactions may mediate maternal exposures that partially reflect the maternal genotype and may occur either before, during or after birth (6). Special analytical approaches are needed to account for the contribution from maternal and paternal parent of origin effects (7, 8).

ADHD management includes pharmacologic and non-pharmacologic treatments. The most widely used drugs in ADHD are amphetamine (used for ADHD since 1937) (9), methylphenidate, and related stimulant and antidepressant drugs, all targeting a small group of monoamine receptors and transporters (10). Diagnoses of psychiatric disorders, including ADHD as described in the Diagnostic and Statistical Manual of Mental Disorders (DSM) (2) and International Classification of Disease (ICD) diagnostic manuals, are mainly based on tradition and practical utility, not on underlying biology. This shortcoming is believed to impede the development of new, effective treatments. In this perspective article, recent progress in ADHD genetics is summarized, with an emphasis on emerging rare variant discovery and its therapeutic implications. The relationship between ADHD and rare Mendelian neurometabolic diseases is reviewed and implications for future research and clinical practice, including “precision psychiatry” is discussed.

## Progress in ADHD Genetics

Early linkage studies in affected families with ADHD produced diverging results. Likewise, candidate studies on genes believed to be implicated in stimulant drug therapy or ADHD symptoms failed to identify major gene effects. Early genome wide association (GWA) studies with modest sample sizes were also inconclusive. Together, these findings indicated that many risk variants and genes may be involved in ADHD etiology (3). The first conclusively genome wide significant findings only emerged after the inclusion of more than 20,000 cases and 35,000 controls (11). Recently, using even larger sample sizes and different statistical methods the number of claimed significant findings have kept growing (12). This pathway of discovery, where the typical sample sizes have increased from hundreds to tens of thousands, resulting in more significantly associated genetic markers, mirrors the development in other genetically complex conditions, including common psychiatric disorders. After reaching a critical minimum sample size, an apparent inflection point is observed where the inclusion of additional samples produce an almost linear increase in significantly associated genetic markers. The exact shape and slope of this path of discovery depends on the genetic architecture of the trait being studied.

Based on the results from GWA studies, a polygenic model of ADHD has emerged, where the genetic risk of ADHD is considered to result from the additive effects many small (or possibly larger) genetic risk variants interacting with each other and with multiple environmental factors. Many of the genetic markers are shared between ADHD and other common psychiatric disorders, such as major depressive disorder (MDD), autism spectrum disorder (ASD) and anxiety disorders (13, 14). This strong genetic relationship is also in accordance with clinical observations showing that ADHD symptoms in children, as well as in adults, often coexists with other symptoms and disorders, including MDD, anxiety and ASD (1). However, despite this progress in ADHD molecular genetics, many questions remain. So far, the discovered genetic variants only explain a small portion of the estimated heritability. The exact role of rare variants and G × E interactions in ADHD is unclear and the clinical implications of the GWAS findings remain to be determined.

## Discovery of ADHD Risk Genes

Single nucleotide variants are the most abundant genetic alteration in the genome. Common single nucleotide variants (>1% allele frequency) are usually termed single nucleotide polymorphisms (SNPs). Genetic variants may have detrimental effects on health and fitness or appear to be functionally silent. The conservation of such SNPs in the population has been partially explained by their low effect on fitness, being relatively protected from the forces of evolution. Typically, such variants are found in non-coding DNA and may be genetically linked to other functional elements or affect gene expression through subtle modification of enhancer region functions. In contrast, the classical Mendelian diseases are typically caused by coding variants with strong effects on fitness and reproduction. However, a closer examination of Mendelian disorders has shown that many have a variable penetrance that is partially dependent on genetic background and presence of other common variants. Indeed, there is no sharp boundary between textbook “genetic” diseases following Mendelian patterns of inheritance and complex conditions. Thus, many common multifactorial, complex human diseases have also been shown to have a combination of genetic risk factors, with some major risk loci in combination multiple other loci of small effects. For example, in Alzheimer's disease (AD), a few common genetic factors, such as the ε4 allele of apolipoprotein E (*APOE*) confers a strong risk. In persons of European ancestry, a 3–4-fold increased risk in heterozygotes and 10–12-fold increased likelihood of AD in homozygotes has been reported (15). However, the observed allele frequencies and relative risks differ in populations of different ethnicities. Similarly, in hypercholesterolemia, which is an important risk factor for atherosclerotic cardiovascular disease and premature death, a few common variants in three genes (*APOB, LDLR, PCSK9*) involved in lipoprotein homeostasis constitutes a large fraction of the total genetic risk (16). The discovery of these risk genes and molecular mechanisms that are attractive targets of intervention has triggered the discovery of new drugs, some of which have been used successfully for many patients.

In comparison, the genetic risk conferred by the individual variants examined in genetic studies of ADHD is typically very small, with odds ratios of 0.835–1.125 for 12 genome wide significant hits reported observed in the first successful ADHD GWA study (11). The genetic prediction offered by each of these variants is minuscule. Even when many genetic risk variants are analyzed together as a polygenic risk score (PRS), the genetic prediction is currently far from being diagnostically useful in clinical settings. This limitation is related to the fact that the susceptibility to a complex trait such as ADHD probably is determined by many specific combinations of genes and environment. The environmental factors are largely unknown and currently used PRSs only capture a fraction of the genetic components, e.g., excluding possible highly penetrant ultra-rare variants not being tagged by common variants. PRS estimates generated in one population also may not transfer well to others, requiring multiple data sources. In conclusion, the polygenic model of ADHD that has emerged from analyzing common risk variants in classical GWA studies only explains a small fraction of total genetic risk in this condition.

Although initial candidate gene studies and linkage studies were difficult to replicate in larger samples, there is emerging evidence that ADHD genetics can also be explained as a combination of different types of genetic variants, ranging from common variants with small effects to rare coding variants with Mendelian patterns of inheritance. Many coding variants in key genes involved in brain development, and function, including neurotransmitter homeostasis have been found in ADHD patients (1, 17, 18). This evidence comes from different sources, including studies on copy number variants (CNVs), large scale exome genotyping and sequencing studies and reports of ADHD co-occurring with classical “genetic” diseases, including neurometabolic diseases. ADHD cases typically have much higher burdens of rare chromosomal duplications and deletions (CNVs) than healthy controls (19). The CNV burden of ADHD cases is similar to that found in autism spectrum disorders (ASD) or schizophrenia (19, 20). Furthermore, recent exome wide genotyping (21) and sequencing and whole genome sequencing (WES/WGS), has shown many ADHD-related rare coding variants with much larger effects (22–26). Many of these variants and genes are established risk genes for other neuropsychiatric disorders. However, due to the rarity of the variants, it is still challenging to obtain the sample sizes needed to formally prove their involvement in ADHD (27).

Progress in pharmacotherapy of ADHD has been slow. Despite decades of basic research and many clinical trials, the most effective and widely used therapies against this condition are still based on the stimulants introduced 50–80 years ago. One of the motivations for conducting genetic studies is to discover causative biological pathways and druggable treatment targets. An examination of the largest GWA study of ADHD conducted so far revealed that none of the significantly associated genes are known targets of currently used drugs against ADHD (28). However, several new, potentially druggable genes/proteins were observed, demonstrating the potential of genetics for discovery of new treatment options.

## Comorbid Conditions

It is well established that ADHD is associated with multiple psychiatric and somatic comorbidities. The latter includes common conditions like obesity, migraine, and asthma (29). In addition, many rare genetic syndromes, including neurometabolic disorders have been associated with ADHD (30). Several inborn errors of amino acid metabolism and transporters appear to converge on common mechanisms that may affect neurotransmitter functions and may be related to co-occurring neuropsychiatric symptoms. For example, inborn errors of aromatic amino acid metabolism in phenylketonuria and tyrosinemia type 1 are characterized by disturbances of dopamine synthesis and symptoms of ADHD (31, 32). Fernandez-Castillo et al. recently conducted a systematic search in the Online Mendelian Inheritance in Man (OMIM) database for Mendelian disorders for genes and diseases presenting with hyperactivity and/or inattention. They identified 139 genes implicated in 137 rare disorders that were mainly related to disturbances of neurodevelopment. Most of these Mendelian disorders were also associated with other psychiatric traits that have been reported together with ADHD (33). This clearly illustrates the diversity of conditions that may cause ADHD symptoms. However, a limitation of this approach is that ADHD symptoms has not been systematically recorded in all Mendelian diseases. Potentially, ADHD symptoms are present in many more diseases than listed in OMIM or similar databases. With the introduction of high throughput molecular genetic diagnostics, the list of rare genetic syndromes that have ADHD symptoms is growing fast.

Some illustrative examples of recent discoveries reported after Fernandez-Castillo et al. conducted their systematic review include patients presenting with ADHD symptoms due to deficiency of L-2-hydroxyglutarate dehydrogenase (*L2HGDH*) (34), X-linked creatine transporter deficiency (35) and *HUWE1* mutations (36). These examples show that severe progressive neurological conditions may masquerade as common ADHD and that clinicians need to be aware of this possibility.

## Other Biomarkers

In addition to genomics, many other types of neurophysiological, brain imaging or biochemical biomarkers have been examined in ADHD (37). Compared to genetic markers, epigenetic, proteomic or metabolomic markers may be more proximally causally linked to function and ADHD symptoms. GWA data may also be combined with brain proteome data to find possible brain proteins altered in ADHD and related psychiatric disorders (38). As an illustration of the potential of this approach, a recent plasma proteomic study of ADHD related biomarkers showed that genetically increased levels of the enzyme beta-mannosidase (MANBA) was associated with a lower risk of ADHD (39). Among the GWAS hits (11) MANBA appeared as one of the druggable proteins (28). Several MANBA inhibitors have been developed as putative treatment against inborn lysosomal storage disorder caused by the deficient activity of beta-mannosidase, but it unclear whether this condition is related to ADHD-symptoms. Still, it shows the relevance of screening patients with neurometabolic diseases for CNS symptoms, including ADHD like symptoms. So far, relatively few non-targeted metabolomic studies on ADHD have been published (37, 40–42). Targeted metabolite studies focusing on pathways considered to be important in brain disorders, such as the kynurenine pathway of tryptophan degradation, have also shown some promising results (43).

## Discussion

Partially inspired from recent progress in cancer treatment, most fields of medicine have experienced an expectation to use individual genetic or other biomarker information for the purpose of patient classification and “individualized,” “precision” or “personalized” medicine. In molecular oncology tumor targeted therapies are based on biomarker results from patient derived cells and tumor tissue. Such stratified treatments have dramatically improved life expectancy for selected groups of patients (44). In comparison, progress in personalized psychopharmacology has been modest. One notable exception involves genotyping of liver catabolizing enzymes, such as CYP2D6 variants that that may predict pharmacokinetic properties and tissue levels of several antidepressants and antipsychotics.

At first glance, ADHD may seem like an ideal candidate for personalized treatments. It is a very heterogenous condition. ADHD symptoms appear with a wide range of severities, clinical manifestations, and patterns of comorbidities. As summarized above, many different rare and common genetic variants and probably also a range of biological mechanisms may produce symptoms of inattention, impulsivity, and hyperactivity. The ideal treatment should be biologically informed and target individual underlying mechanisms and risk factors. However, in clinical practice, pharmacotherapy of ADHD is still largely based on trial and error with titration of broadly acting stimulant drugs affecting common neuromodulating monoamine transmitters. Although the majority of patients show some symptom reduction from stimulant drug therapy, many experience side effects or have no clear benefit from these drugs. Although several pharmacogenetic tests have been suggested, mainly targeting polymorphisms in dopamine and noradrenaline transporters or receptors, as well as stimulant metabolizing enzymes such carboxylesterase 1, there are currently no generally accepted reliable genetic or other biomarkers tests that can predict treatment outcome in this patient group (45).

Different scenarios may be imagined for the introduction of genome guided personalized therapies for ADHD. It is theoretically possible that pharmacogenomics might reveal common, converging biological pathways, including new druggable targets for large patient groups. However, as mentioned above, ADHD appears to be a highly heterogenous condition, involving many additive and interacting genetic and environmental risk factors. Thus, a more realistic option is that personalized ADHD therapies will only be introduced gradually for small patient groups where ADHD symptoms might be secondary e.g., to defined neurometabolic aberrations. This expectation is based on experiences from oncology and other therapeutic areas, where personalized treatments have been introduced very slowly in based on the identification of particular biological signatures that are present only in a small minority of patients.

Genetic research has clearly shown that each person with ADHD is unique. However, before personalized ADHD therapies can become a clinical reality, there are many challenges and knowledge gaps that need to be addressed. First, the whole spectrum of risk factors and biological mechanisms of ADHD needs to be systematically explored. This will require much larger sample sizes than currently available, defined clinical subgroups and the merging of data from studies on common and rare genetic variants. Biologically meaningful subgroups may need to include patients across traditional diagnostic categories. In addition to genomics data, information from multiple data sources, including epigenetics, transcriptomics, metabolomics and proteomics need to be integrated and jointly analyzed to describe biological profiles in these subgroups. Such -omics and systems medicine approaches are essential for identifying the relevant patients, whether they are suffering from a potentially treatable cancer or a progressive brain damaging neurometabolic disease.

Given that sufficient research progress can be made, the practical implementation of genome guided therapies may be equally challenging. Clinicians need to be aware of new therapeutic possibilities, to be trained to recognize relevant symptoms and to have access to adequate laboratory resources, including a medical genetics/ systems biology service. As detailed above, in cases with atypical or progressive clinical features and co-occurring somatic manifestations it is vital to properly explore possible underlying conditions and to examine for coexisting problems beyond classical ADHD symptoms. In conclusion, as research on ADHD genetics is coming of age, personalized (drug) therapy could become a reality also for ADHD patients. This could start with the most obvious genetic perturbations affecting known targets of currently used drugs, as well as new druggable targets identified using pharmacogenetic pipelines of discovery.

## Data Availability Statement

The original contributions presented in the study are included in the article/supplementary material, further inquiries can be directed to the corresponding author/s.

## Author Contributions

The author confirms being the sole contributor of this work and has approved it for publication.

## Funding

This study has received funding from the Research Council of Norway (Project No. 331725), Stiftelsen Kristian Gerhard Jebsen (SKJ-MED-02), the Regional Health Authority of Western Norway (25048), and the European Union's Horizon 2020 Research and Innovation Programme under Grant Agreement No. 728018.

## Author Disclaimer

This article reflects only the author's views and the European Union is not liable for any use that may be made of the information contained therein.

## Conflict of Interest

JH has received lecture honoraria as part of continuing medical education programs sponsored by Shire, Takeda, Medice, and Biocodex.

## Publisher's Note

All claims expressed in this article are solely those of the authors and do not necessarily represent those of their affiliated organizations, or those of the publisher, the editors and the reviewers. Any product that may be evaluated in this article, or claim that may be made by its manufacturer, is not guaranteed or endorsed by the publisher.
